# Advanced Tree-Based Techniques for Predicting Unconfined Compressive Strength of Rock Material Employing Non-Destructive and Petrographic Tests

**DOI:** 10.3390/ma16103731

**Published:** 2023-05-15

**Authors:** Yuzhen Wang, Mahdi Hasanipanah, Ahmad Safuan A. Rashid, Binh Nguyen Le, Dmitrii Vladimirovich Ulrikh

**Affiliations:** 1 School of Civil Engineering, Henan Vocational College of Water Conservancy and Environment, Zhengzhou 450008, China; 2 School of Water Conservancy and Civil Engineering, Zhengzhou University, Zhengzhou 450001, China; 3 Institute of Research and Development, Duy Tan University, Da Nang 550000, Vietnam; 4 Faculty of Civil Engineering, Universiti Teknologi Malaysia, Johor Bahru 81310, Malaysia; 5 School of Engineering & Technology, Duy Tan University, Da Nang 550000, Vietnam; 6 Department of Urban Planning, Engineering Networks and Systems, Institute of Architecture and Construction, South Ural State University, Lenin Prospect 76, 454080 Chelyabinsk, Russia

**Keywords:** rock strength prediction, physical properties, non-destructive tests, regression tree techniques, gradient boosting tree, random forest

## Abstract

The accurate estimation of rock strength is an essential task in almost all rock-based projects, such as tunnelling and excavation. Numerous efforts to create indirect techniques for calculating unconfined compressive strength (UCS) have been attempted. This is often due to the complexity of collecting and completing the abovementioned lab tests. This study applied two advanced machine learning techniques, including the extreme gradient boosting trees and random forest, for predicting the UCS based on non-destructive tests and petrographic studies. Before applying these models, a feature selection was conducted using a Pearson’s Chi-Square test. This technique selected the following inputs for the development of the gradient boosting tree (XGBT) and random forest (RF) models: dry density and ultrasonic velocity as non-destructive tests, and mica, quartz, and plagioclase as petrographic results. In addition to XGBT and RF models, some empirical equations and two single decision trees (DTs) were developed to predict UCS values. The results of this study showed that the XGBT model outperforms the RF for UCS prediction in terms of both system accuracy and error. The linear correlation of XGBT was 0.994, and its mean absolute error was 0.113. In addition, the XGBT model outperformed single DTs and empirical equations. The XGBT and RF models also outperformed KNN (R = 0.708), ANN (R = 0.625), and SVM (R = 0.816) models. The findings of this study imply that the XGBT and RF can be employed efficiently for predicting the UCS values.

## 1. Introduction

In various domains of geotechnical engineering structures, including tunnels and dams, appropriate measurement of unconfined compressive strength (UCS) of rocks is of vital significance. The UCS provides a desirable evaluation of the capacity of rock bearing. To be specific, an unsuitable calculation of the UCS can be dangerous, because it causes the depreciation of the final bearing capacity. The unconfined compression test in labs is typically employed to ascertain rock strength. The standard procedures, including the International Society for Rock Mechanics (ISRM), are followed to carry out the test. Nevertheless, various hindering issues in the direct determination of UCS in the lab are available. For example, providing the needed rock core specimens is usually challenging, particularly for highly fractured rocks and those that show notable foliation and lamination [1,2]. It is extremely costly and prolonged for ascertaining the UCS directly at the initial steps of design [3]. Although, different and secondary methods of rock strength prediction are available, including common regression models and machine learning techniques.

To date, many researchers have sought to develop standard techniques for UCS assessment. Certain distinct techniques for UCS forecast are regularly in the classes of simple regression between UCS and straightforward index tests of rocks, including the Schmidt hammer, ultrasonic velocity, or Vp, Brazilian tensile strength, point-load index, and slake durability index tests [4,5,6]. In addition, the successful utilisation of multiple regression analysis for rock strength forecast is also reported in the literature [7]. However, some available reports found that these associations were inadequate for producing extremely reliable UCS values [8,9]. Typically, it is suggested to employ these equations simply for particular types of rock [9]. Moreover, the analytical forecast techniques cannot adjust themselves with the changes in data, which means if different data from the initial set of data is added to these models, the equations should be renewed [10,11]. More recently, researchers and practitioners of geotechnical engineering highlighted the utilisation of machine learning (ML) techniques, including decision trees (DTs), the artificial neural network (ANN), support vector machine (SVM), and the adaptive neuro-fuzzy inference system (ANFIS) in problems of this domain [12,13,14,15]. These highlighted techniques are related to the firm fact that ML techniques are fit and likely instruments for engineering problem-resolving, especially when the association varieties between the predictors and target variable are concealed [11,16,17,18,19,20,21,22,23,24,25,26]. From a monetary perspective, the application of ML techniques is also profitable, because it reduces the costs related to lab tests for ascertaining the UCS. It is important to note that the mentioned ML techniques have been used and applied to solve science and engineering problems [21,23,27,28,29,30,31,32,33,34,35,36,37,38,39,40,41,42,43,44,45,46,47,48,49,50,51,52,53,54,55,56,57,58,59].

The UCS value of the Main Range granite in Malaysia is predicted in this work using two state-of-the-art tree-based techniques, namely random forest (RF) and extreme gradient boosting trees (XGBT). These two models are among the most robust ML models, and their predictive potential has been shown in several areas of study, e.g., [60,61]. To date, however, these approaches have not been used to forecast the unconfined compressive strength of rock materials. These models are also less susceptible to overfitting, a major problem with ML approaches with limited data (which is also true for this study). This process considers and selects non-destructive rock index tests and petrographic investigations, as both categories are crucial in predicting rock strength values. Comparative analysis is also conducted among the RF and XGBT models, simple regression, and single DTs.

## 2. Rock Strength Research Significance

In the past, rock strength was considered as the model output of many empirical, semi-empirical, and intelligent studies. In these studies, both destructive parameters such as point load index and Brazilian tensile strength (BTS) and non-destructive parameters such as p-wave velocity and Schmidt hammer have been considered as model inputs. However, conducting some of these tests, such as BTS, is still time-consuming and requires sample preparation. On the other hand, rock minerals have theoretical relationships with strength-related parameters of the rock material, and they can also be considered as input parameters to predict rock strength. These minerals and their percentages can be easily identified through petrographic analysis on thin sections of the same rock. There are only a few studies considering different rock minerals as input variables, and there is a need to combine these parameters with non-destructive parameters as inputs to estimate rock strength. This can aid in the development of a predictive model with simpler input parameters and, as a result, greater applicability in real-world projects.

## 3. Earlier Related Studies

Several previous studies employed ML techniques for UCS prediction. In 1999, a study by Meulenkamp and Grima [62] forecasted UCS was utilising a backpropagation ANN. These researchers applied this technique to 194 various kinds of rock samples, including dolomite, sandstone, and limestone. They have employed several inputs for predicting the UCS, including density, porosity, grain size, rock type, and Equotip hardness reading. Their findings showed that the ANN outperformed the traditional statistical techniques in terms of generalization capabilities. A fuzzy inference system (FIS) was applied to 164 samples of Ankara agglomerate by Sonmez et al. [63] for the UCS forecast. Their results indicated that the fuzzy logic technique yields a highly reliable UCS prediction. A different study by Gokceoglu and Zorlu [64] applied both regression and fuzzy models to 82 samples from problematic rocks for UCS prediction. They considered the ultrasonic velocity, point load index, tensile strength, and block punch index as their inputs. In comparison with multiple and simple regression methods, the fuzzy model had a better performance for UCS prediction. Feed-forward neural network and regression models were applied to a dataset including 30 travertine rock data by Dehghan, Sattari, Chelgani, and Aliabadi [8]. Their inputs included Schmidt hammer rebound number, velocity, porosity, point load index, and ultrasonic. This study suggested that the ANN method is a more powerful model than regression analysis for UCS prediction. Mishra and Basu [65] employed simple and multiple regression techniques as well as FIS. They suggested that the FIS and multiple regression techniques are more efficient for predicting the UCS than the simple regression technique. For sedimentary rock samples, the efficiency of ANN was reported by Cevik et al. [66]. They applied this model to 56 samples and considered clay contents, the origin of rocks, slake durability indices, two/four-cycle as inputs of UCS prediction. A different study by Yesiloglu-Gultekin, Gokceoglu, and Sezer [18] reported the advantage of the ANFIS over the multiple regression and ANN. They employed 75 rock samples from a granite mine. They utilized p-wave velocity, rock tensile strength, block punch index, and point load index as inputs. They also concluded that if p-wave velocity as well as tensile strength are considered for building the ANFIS prediction model of UCS, this model shows its best performance.

Singh et al. [67] found a number of relationships between various measures of strength and various schistose rock index characteristics. They also applied an ANN to their dataset and concluded that the ANN outperformed the established correlations in terms of accuracy. Gokceoglu [68] employed a fuzzy triangular chart for UCS prediction relating to the petrographic composition. The author created fifteen membership functions for fifteen samples. It was demonstrated that the fuzzy inference system could reliably predict the UCS values. Zorlu et al. [69] examined the associations between UCS and petrographic properties of sandstone. They evaluated the accuracy of multiple regression and ANN for the prediction of stone UCS. Their conclusion showed the superiority of ANN over multiple regression. For UCS forecast of carbonate rocks, Yagiz et al. [70] developed nonlinear regression and ANN models. Their dataset included 54 samples. They also stated that the ANN model outperforms the nonlinear regression model in terms of accuracy. Table 1 presents a summary of some recent studies on the application of ML techniques for predicting the UCS.

## 4. Collection of Case Studies and Data

The team of this research collected the rock samples from a water transfer tunnel, starting from Pahang state and ending in Selangor state. The data were collected from the study published by Armaghani et al. [73]. The tunnel supplies the extra water needs for Kuala Lumpur and Selangor states. The tunnel was constructed by the tunneling workforce to pass through the Main Range, which runs between the states of Pahang and Selangor. The spine of Peninsular Malaysia with a height range of 100 to 1400 m is formed by this mountain chain. Granite is the major type of rock, which is locally called Main Range granite. The intact granite strength is typically between 150 and 200 MPa. Table 2 shows the specifications of the tunnel.

Thirty-five kilometers of the tunnel were unearthed utilizing a tunnel boring machine (TBM). The rest of the tunnel was unearthed employing traditional tools and blast techniques. For getting the best excavation performance for this tunnel, the tunnelling team planned three TBM and four traditional drill and blast segments.

The research team collected a number of core samples from the boreholes and sent them to a laboratory to check the important granite material and engineering attributes. The team also lapped the edge of the surfaces of the cut cores to achieve the needed finishing and appearance. For the laboratory tests, many granitic rock material samples were prepared. To avoid any unwanted differences in features and early breakdowns, the team carefully inspected the samples for extant fractures and different tiny-scale discontinuities. The physical characteristics (i.e., non-destructive) tests involved Vp and dry density (DD). In addition, uniaxial compression examinations were conducted to ascertain the granite UCS. The team followed the ISRM [74] standards of testing and preparing all tests and investigations. Eventually, a database with the 45 data samples (all input and output parameters) was prepared for data analysis.

A polarizing petrological microscope was also used to conduct the petrographic investigations of the granite samples. To this end, small segments of the samples were provided to distinguish the portion of various minerals. The specimens display non-porphyritic and holocrystalline mineral composition as well as essentially comprise interlocking coarse-grained crystals of quartz (Qtz), plagioclase (Plg), biotite (Bi), and alkali feldspar (Kpr). The before-mentioned composition is expected of plutonic igneous rock. It is important to note that micas are denoted by 3 elements: sericite, muscovite, and biotite. For more information regarding study area and the data used for modeling, it is recommended to review Armaghani, Mohamad, Momeni, and Narayanasamy [73].

## 5. Analysis of Data

**Figure 1:**
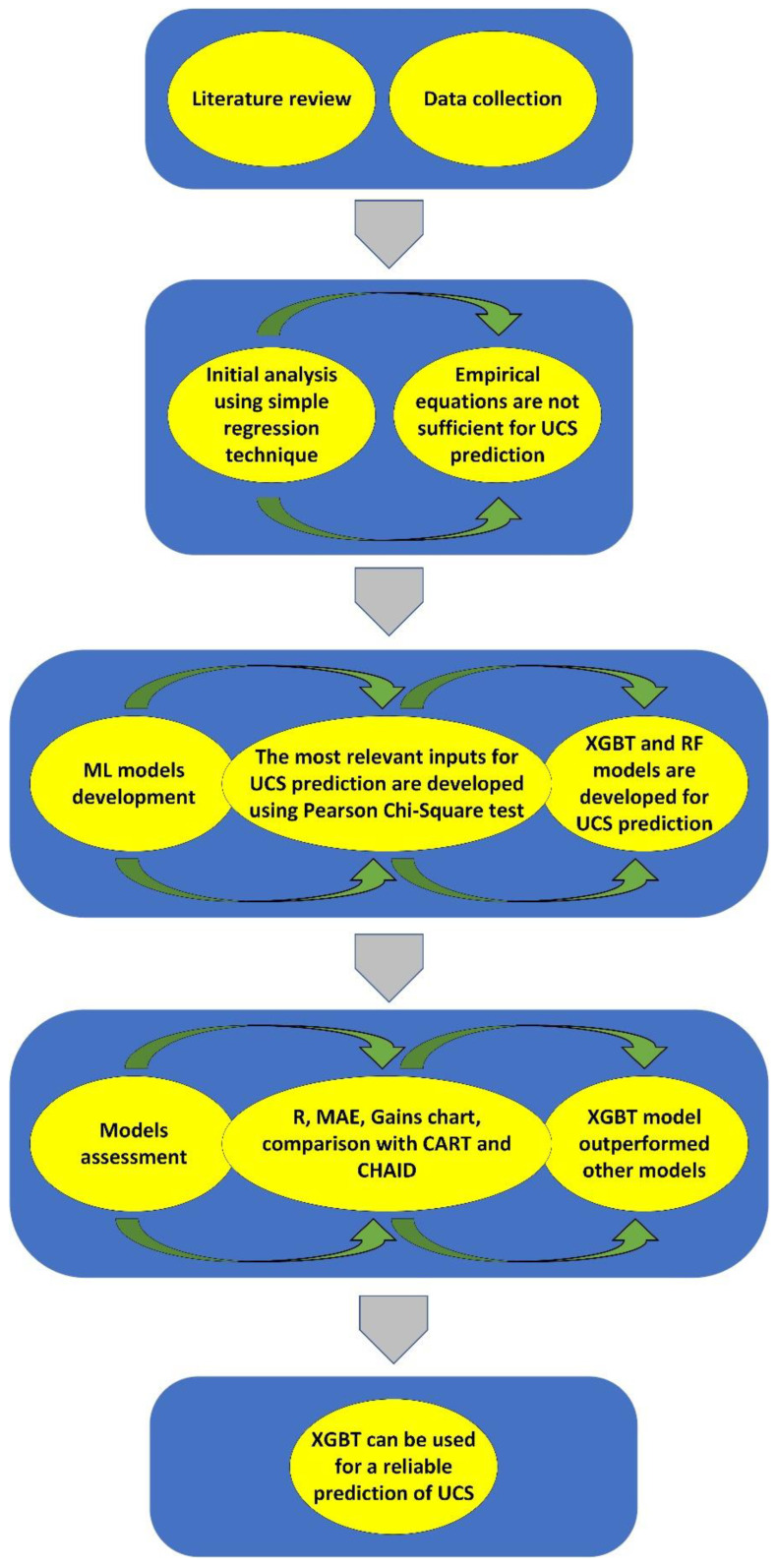
Process of this study.

In this study, various statistical and simulation techniques were employed to analyze the results of laboratory tests. The subsequent sections explain the application of the techniques mentioned before to forecast the UCS of granite samples. Ultimately, the UCS values achieved from laboratory tests were compared with those predicted. The process of this study is presented in Figure 1. Two ML algorithms, including XGBT and RF, were employed to predict the UCS. Before the models’ development, an input selection was performed using the Pearson’s Chi-Square test. The inputs selected by this test were used to develop XGBT and RF models. The models were then evaluated by several performance criteria, including R, MAE, and gain charts. Additionally, the results of XGBT and RF models were compared with those of single decision trees, including CART and CHAID.

### 5.1. Regression Techniques

**Figure 2:**
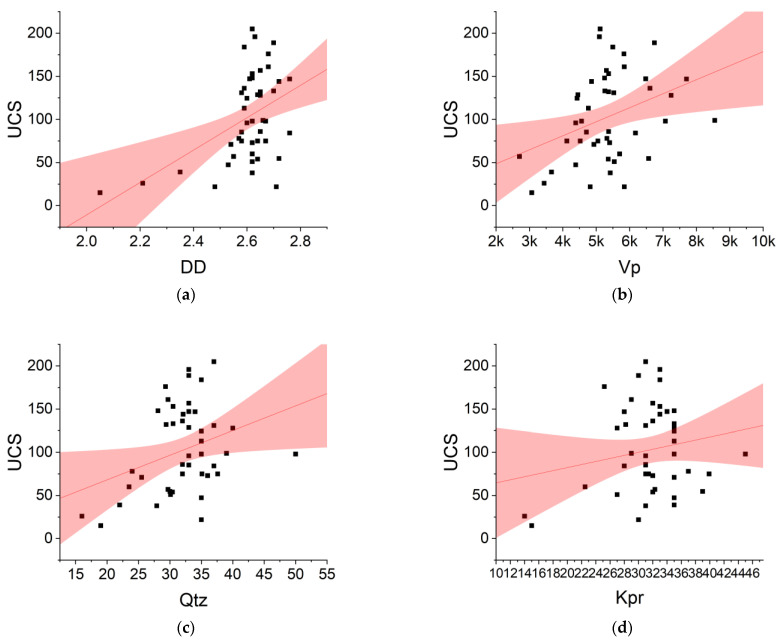

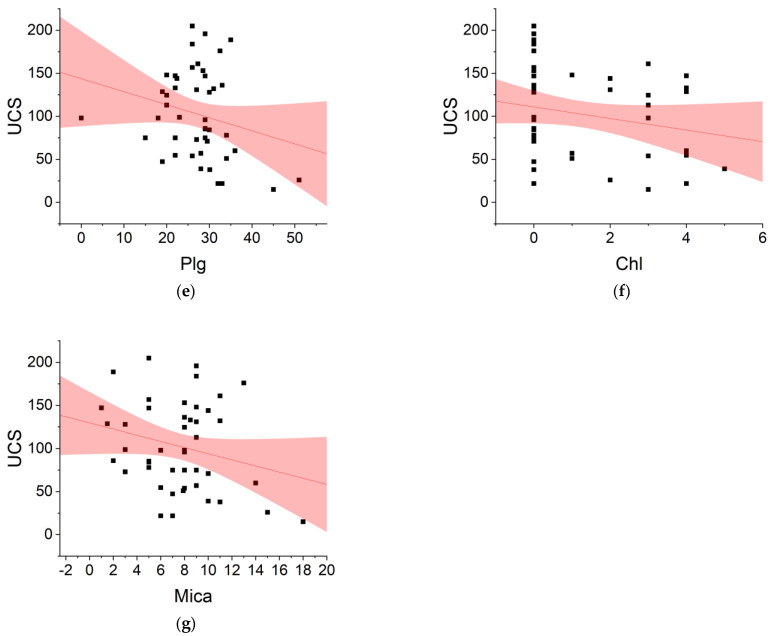
Simple empirical regression models for predicting UCS. (**a**) UCS = 187.66 × DD − 386.02; R^2^ = 0.2136; (**b**) UCS = 0.0163 × Vp + 15.989; R^2^ = 0.1301; (**c**) UCS = 2.8535 × Qtz + 11.013; R^2^ = 0.0998; (**d**) UCS = 1.7699 × Kpr + 46.883; R^2^ = 0.0342; (**e**) UCS = −1.5152 × Plg + 143.7; R^2^ = 0.0539; (**f**) UCS = −6.718 × Chl + 110.84; R^2^ = 0.0469; (**g**) UCS = −3.563 × Mica + 129.63; R^2^ = 0.0611.

This study developed several empirical equations using simple regression technique. The simple regression is represented by the general form of Y = AX + B, “A” is the coefficient of regression and “B” is a constant value of “Y” once all input parameters equal zero. These equations and their square of the correlation coefficient (R^2^) are shown in Figure 2. Among all variables used in this study, DD was the most correlated variable to UCS, followed by Vp. As can be seen, the best R2 belonged to Figure 2a followed by Figure 2b. The lowest R^2^ belonged to Figure 2d. However, these accuracies show that empirical regression models applied to only one predictor are insufficient to solve the UCS issues. According to Yilmaz and Yuksek [7], the main conceptual shortcoming of all regression approaches is that they cannot reveal the precise causation process, just connections.

Using their assumption-free characteristics, ML approaches may circumvent the above difficulties. There are several ML approaches capable of resolving UCS-related issues. Among these ML approaches, academics and practitioners paid less attention to DTs and their ensemble variations. This work will thus use two powerful ensemble DT methods, RF and XGBT, to forecast the UCS values of Malaysian Main Range granite.

### 5.2. Decision Tree Models

Various decision tree (DT) models are available and each of them has its own advantages and disadvantages. DTs employ a tree-like model of choices and their potential outcomes, such as chance event results, resource costs, and utility. It can show a conditionally controlled scheme. Some well-known DTs are classification and regression tree (CART), Chi-square automatic interaction detection (CHAID), and quick, unbiased, efficient statistical tree (QUETS). However, these tree-based models are classified as single DTs. The common flaw of these single DTs is their susceptibility to overfit with a small training set.

**Figure 3:**
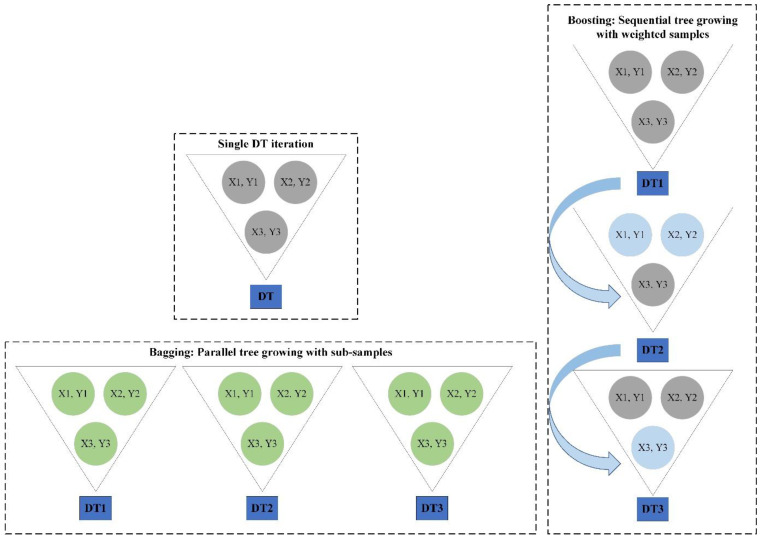
Application of bagging and boosting in DTs.

To remedy the abovementioned issue, some other advanced DTs, including random forest (RF) [75] and extreme gradient boosting tree (XGBT) [76] can be employed. Both algorithms belong to the ensemble DTs family. While RF follows the bagging principles, the XGBT follows the boosting standards. A schematic diagram of boosting and bagging and their application in DTs is presented in Figure 3.

RF is a variance-reducing bagging method, as was previously explained. DTs are very sensitive to even little variations in the input. By using the “bagging” method, the RF is able to generate a stable model that lessens the variation. Bagging is an ensemble method that uses several averaging procedures, such as the mean, the majority vote, and the weighted average, to construct and integrate many predictions.

One of the most efficient machine learning techniques for classification and regression problems is gradient boosting (GB). GB creates a strong predictive model by combining weak learners such as DTs. If the weak learner is a DT, the resulting model is termed a gradient boosted tree (GBT). The GBT employs a conventional technique, including mean squared error (MSE), to build the regression trees and ascertain the most desirable division for the tree. For each possible node division, the GBT approximates the MSE and selects the one with the lowest MSE as the split to utilize in the tree.

One of the variants of GBT, which employs more precise estimates to decide the most suitable tree model, is Extreme Gradient Boosting Tree (XGBT). The XGBT applies several efficient methods, which make it extraordinarily strong, especially with structured data. Contradictory to conventional GBT, XGBT applies its process of creating trees where the similarity score (SS) and gain ascertain the fittest node divisions. The SS can be calculated utilizing Equation (1). Once the SS for each leaf is calculated, the gain can be estimated using Equation (2). Then, the node division with the greatest gain is selected at the most suitable division for the tree.

(1) $$\mathrm{SS}=\frac{{\left(\sum_{i=1}^{n}R_{i}\right)}^{2}}{\sum_{i=1}^{n}\left[{\mathrm{PP}}_{i}*\left(1-{\mathrm{PP}}_{i}\right)\right]+\lambda }\;$$

(2) $$G={\mathrm{LL}}_{s}+{\mathrm{RL}}_{s}-{\mathrm{RO}}_{s}$$

where SS is the similarity score, “R” represents the residual, PP signifies previous probability, λ denotes a regularization parameter. LLs is the left leaf similarity, RLs is the right leaf similarity, ROs is the root similarity.

## 6. Results and Discussion

This research applied two advanced decision tree techniques, including RF and XGBT, to predict the UCS. These techniques were applied to 45 data points or samples collected from the case study sites. Before the development of these models and because the data were unbalanced, the input and target fields were normalized. For this purpose, a z-score transformation technique was utilized for inputs and a Box–Cox transformation technique was used for the target variable.

In order to reduce the data dimensionality and identify the most relevant predictors of UCS prediction, the authors employed a Pearson’s Chi-Square test. The candidate inputs included DD, Vp, Qtz, Kpr, Plg, Chl, and Mica. The feature selection technique selected the most important predictors, including DD, Vp, Qtz, Plg, and Mica, which are used for developing the RF and XGBT models.

**Figure 4:**
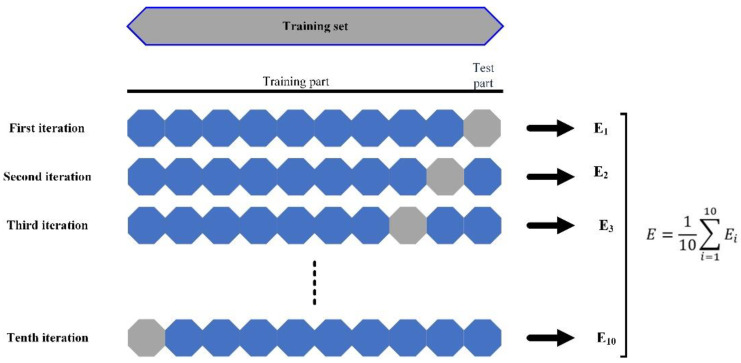
A ten-fold cross-validation procedure.

To develop the RF and XGBT models, 10-fold cross validation was used as the validation scheme. Since there are few samples, as a rule of thumb, the cross validation works better than the holdout technique. Cross-validation protects the ML predictive models against overfitting, especially when the number of samples is limited. This technique divides the sample frame into an established number of parts, and then averages the overall errors. The cross-validation procedure is shown in Figure 4.

**Figure 5:**
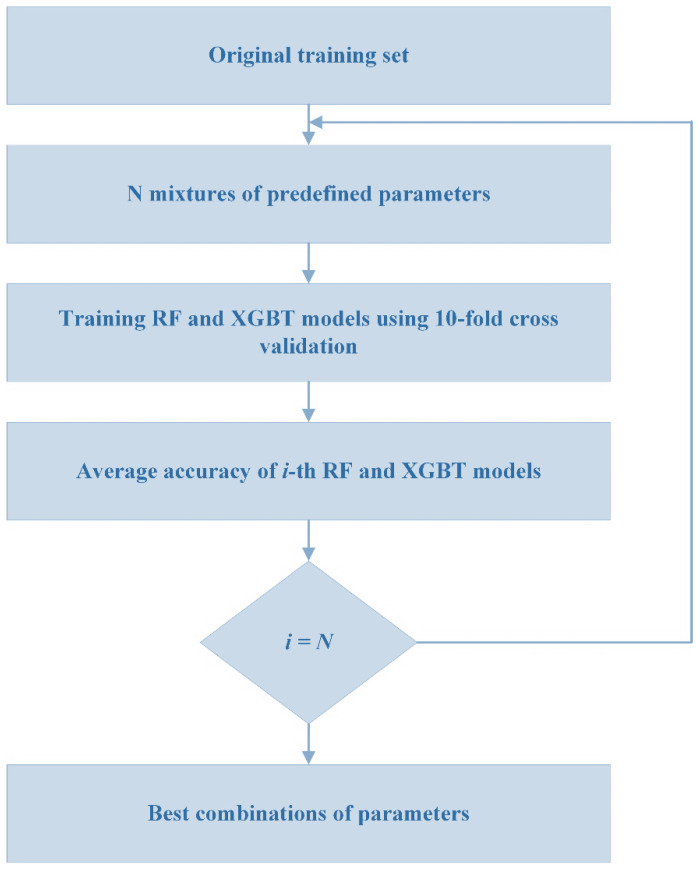
Process of grid search in this study.

The RF and XGBT models were developed using several parameters. To achieve the optimized value for each parameter of these models, a grid search technique was used. This technique systematically creates and assesses a model for various mixtures of algorithm parameters particularized in a grid. Figure 5 displays the process of the grid search technique while a 10-fold cross validation technique is applied.

For RF, the following parameters were optimized and used: (1) number of trees to build = 10.0; minimum leaf node size = 1.0. The algorithm also used bootstrap and out-of-bag samples to calculate the accuracy of generalization. For XGBT, (1) the booster type was set as “gbtree”; (2) the boosting round number was set as 10; (3) Lambda was set as 1.0; (4) Alpha was set as 0.0; (5) max depth was set as 6.0; and (6) minimum child weight was 1.0.

Two standard metrics, linear correlation (R) and mean absolute error (Mae), were used to evaluate the effectiveness of these models (MAE). Below are the equations for determining these criteria:

(3) $$R=\frac{\sum_{i=1}^{n}\left(e_{i}-{\bar{e}}_{i}\right)\left(m_{i}-{\bar{m}}_{i}\right)}{\sqrt{\sum_{i=1}^{n}{\left(e_{i}-{\bar{e}}_{i}\right)}^{2}{\left(m_{i}-{\bar{m}}_{i}\right)}^{2}}}$$

(4) $$\mathrm{MAE}=\frac{\sum_{i=1}^{n}\left|e_{i}-m_{i}\right|}{n}$$

where e_i_ and m_i_ denote nth real and predicted values, respectively; ${\bar{e}}_{i}$ and ${\bar{m}}_{i}$ signify the average values of actual and forecast values, correspondingly; n stands for the number of samples in the dataset.

**Figure 6:**
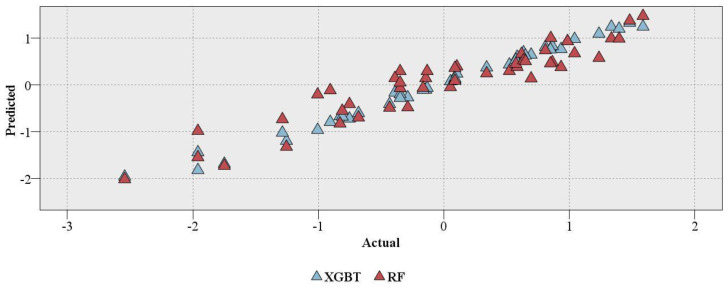
Real vs. predicted values of XGBT and RF.

The RF and XGBT models were evaluated with the abovementioned statistical evaluations (Figure 6) with further assessments of minimum, maximum, and mean errors (Table 3). The evaluation of models developed in this study showed a more accurate prediction of XGBT as compared to the RF model. The R of XGBT and RF models were 0.994 and 0.939, respectively. In addition, the MAE of XGBT and RF models were 0.113 and 0.298, respectively. A plot of actual and predicted values of XGBT and RF models is shown in Figure 6.

**Figure 7:**
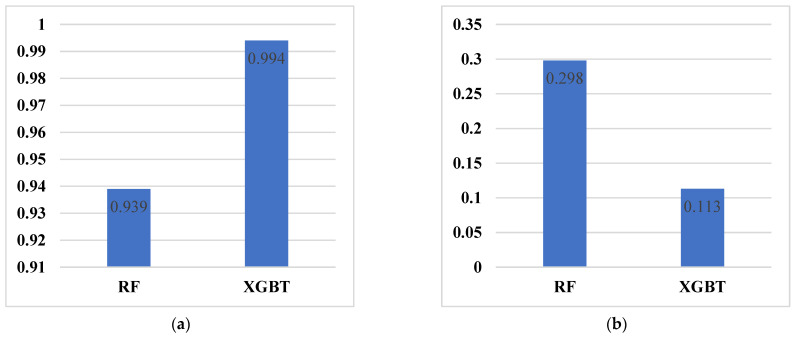
The performance of the RF and XGBT models. (**a**) R; (**b**) MAE.

**Figure 8:**
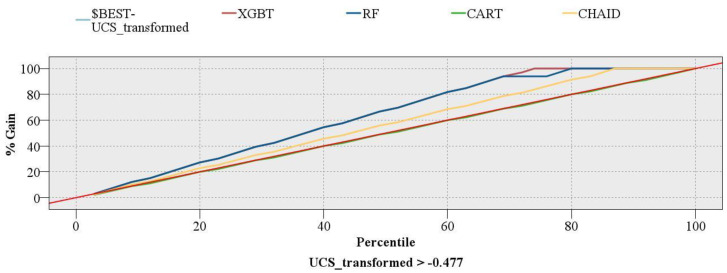
Gains chart of the models developed in this study.

Two single DT models, CART and CHAID, which can be used for regression problems, were also developed to compare these models with XGBT and RF. The R of CART and CHAID models were zero and 0.65, respectively. For MAE, CART and CHAID had values of 0.811 and 0.593, respectively (Figure 7). In addition, a gains chart provides a more in-depth comparison between these models (Figure 8). In machine learning, the gains are explained as the proportion of entire hits that happen in every quantile. The gains are estimated using Equation (5). Here, “hits” mean how the model is successful in forecasting the values greater than the middle value of the UCS (UCS_transformed > −0.477).

(5) $$\mathrm{Gains}=\frac{a}{b}\times 100$$

where “a” implies the number of hits in quantile and “b” refers to the aggregate number of hits.

In Figure 8, the red diagonal line shows the baseline, and the pale blue line represents the perfect model. Typically, the higher lines show better models. As can be seen, the lowest line belongs to CART and follows the diagonal line from the lower left to the upper right. It is evident from the chart that RF and XGBT models have better performance than the other two single DT models. More importantly, the XGBT model followed the perfect model, which shows the superiority of this model over the RF and other models.

The better performance of XGBT over the RF can have several reasons. To start, XGBT has a high similarity score for a direct pruning tree, which is a first step toward the ultimate modeling objectives. Selecting the right node and figuring out the information gain is made simpler by the similarity score. Second, XGBT is trustworthy when working with imbalanced datasets, but RF is not. One last key distinction between XGBT and RF is that the former routinely gives more weight to functional space when reducing a model’s cost, while the latter aims to allow greater leeway to hyperparameters to improve the model.

**Figure 9:**
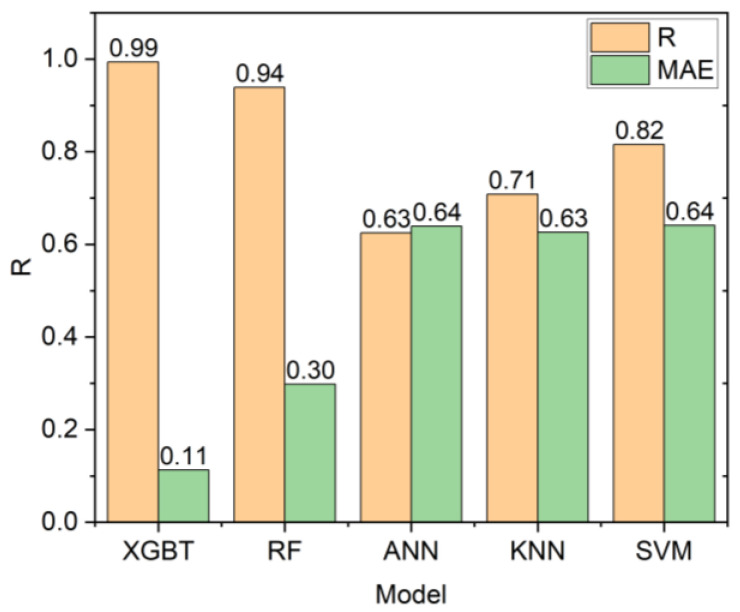
Models’ comparison.

This study also compared the results of the models developed in this study with those of some other common ML techniques, including k-nearest neighbors (KNN), ANN, and SVM. The outcomes of this comparison are shown in Figure 9. As can be seen, both XGBT and RF outperformed KNN, ANN, and SVM models.

When comparing the XGBT model’s accuracy (R = 0.994) to that of prior research, we found that Armaghani, Mohamad, Momeni, and Narayanasamy [73], who used an ANFIS model with the identical sample size and four inputs, performed somewhat better. Furthermore, the accuracy attained in this investigation was better than that of previous studies shown in Table 1.

## 7. Limitations and Future Works

It is a common fact that ML studies have always included several limitations and difficulties. One of the limitations of this study is related to the rock type, which is granite. The next limitation is related to the number of data samples used in the analysis, which is 45. The proposed models in this research are effective with the expected accuracy if the same input parameters are used in the future. In addition, if the same inputs are used but out of the range of our inputs, there is a possibility of an error in the analysis. Future studies with more data samples (more petrographic tests) should be conducted to propose a more generalized ML model. Furthermore, additional rock index tests, such as point load tests, should be performed and used as model inputs to map the behaviors of rock strength in greater and more accurate detail. 

## 8. Conclusions

This present study aimed to predict the UCS using two advanced ensemble tree-based models, including the RF and XGBT. These models were applied to 45 data samples of rock material collected from a mountain range in Malaysia. Before the development of these two models, some simple regression equations were developed and showed that these models were insufficient for UCS prediction. Among the candidate inputs, the Pearson’s Chi-Square test selected the following as the inputs of the XGBT and RF models: DD, Vp, Qtz, and Plg. These two models were developed, and the results showed that the XGBT model outperformed the RF in terms of both accuracy and error. The XGBT model had a linear correlation of 0.994, while the RF model had a linear correlation of 0.939. These models were compared with two single DTs, including CART (linear correlation = 0) and CHAID (linear correlation = 0.65). Expectedly, both ensemble models outperformed the single DTs. In addition, XGBT and RF models were compared with ANN, KNN, and SVM models. Again, the two ensemble models outperformed the ANN (linear correlation = 0.625), KNN (linear correlation = 0.708), and SVM (linear correlation = 0.816) models.

The sample size of this study was small. Further studies can apply these advanced ensemble DT techniques to larger sample sizes to achieve higher accuracies. However, depending on the specifics of the situation, the aforementioned predictive methods may be used to make predictions about UCS. Furthermore, it was said that the use of XGBT and RF offers a practical method for reducing uncertainties during the design of rock engineering projects, which is important in terms of performance. Many additional mining difficulties, not only Young’s modulus, may be predicted using these approaches. It has been shown that XGBT is a practical, accurate, and effective algorithm. Tree learning techniques and linear model solvers are both in XGBT’s toolkit. Being able to conduct several computations in parallel at once is what makes it so fast.

## Figures and Tables

**Table 1:** Some studies on UCS forecast utilizing ML techniques.

Author	ML Technique	Input	R
Beiki, Majdi and Givshad [9]	GP	Density, p-wave velocity, porosity	0.91
Ceryan et al. [71]	ANN	Porosity, slake durability index, p-wave velocity in solid part of the sample, effective porosity, petrography study values	0.94
Dehghan, Sattari, Chelgani, and Aliabadi [8]	ANN	P-wave velocity, slake durability index, porosity	0.93
Gokceoglu and Zorlu [64]	FIS	Slake durability index, block punch index, p-wave velocity, BTS	0.82
Meulenkamp and Grima [62]	ANN	Equotip value, porosity, density, grain size	0.97
Momeni, Nazir, Armaghani, and Maizir [20]	PSO-ANN	Density, p-wave velocity, slake durability index,	0.98
Rabbani et al. [72]	ANN	Porosity, bulk density, water saturation	0.98
Rezaei, Majdi, and Monjezi [11]	FIS	Density, porosity	0.97
Singh, Singh, and Singh [67]	ANN	Petrography study values	-
Marto, Hajihassani, Jahed Armaghani, Tonnizam Mohamad, and Makhtar [19]	PSO-ANN	Slake durability index, BTS, bulk density, p-wave velocity	0.98
Yesiloglu-Gultekin, Gokceoglu, and Sezer [18]	ANFIS	BTS, p-wave velocity	0.77
Yilmaz and Yuksek [7]	ANFIS	P-wave velocity, slake durability index, water content	0.97
Zorlu, Gokceoglu, Ocakoglu, Nefeslioglu, and Acikalin [69]	ANN	Quartz content, packing density, concavo convex	0.87

**Table 2:** Tunnel specifications.

Specification	Value
Length	44,600 m
Diameter	5.2 m
Longitudinal gradient	1/1.900
Maximum discharge of raw water	27.6 m^3^/s

**Table 3:** Statistical error evaluation functions of the models.

	RF	XGBT
Minimum error	−0.975	−0.58
Maximum error	0.658	0.34
Mean error	−0.062	−0.041

## Data Availability

The data will be available from the corresponding author upon reasonable request.
